# Investigating GDS and its symptoms with ATN biomarkers in diverse populations

**DOI:** 10.1002/alz70856_099096

**Published:** 2025-12-24

**Authors:** Gita Pathak, Kumudu Subasinghe, Robert Barber, Nicole Phillips

**Affiliations:** ^1^ Icahn School of Medicine at Mount Sinai, New York, NY, USA; ^2^ University of North Texas Health Science Center, Fort Worth, TX, USA; ^3^ Department of Microbiology, Immunology and Genetics UNT Health Science Center, Fort Worth, TX, USA

## Abstract

**Background:**

Depression, particularly in late life, is associated with an increased risk of Alzheimer's Disease (AD), yet its underlying biological link to AD pathology remains unclear. Investigating plasma biomarkers in individuals with depressive symptoms may help determine whether these symptoms reflect early AD‐related neurodegeneration or an independent neuropsychiatric process. Therefore, we aim to investigate how plasma biomarkers of AD can provide critical insights into the overlapping neurobiological mechanisms between depression and AD.

**Method:**

We leverage data collected from participants in the Texas Alzheimer's Research Care Consortia (TARCC). Recent depression history was collected as part of medical history, and participants were assessed for depression symptoms using a 30‐question model, with a total score collected for each participant, called the Geriatric Depression Scale (GDS). We limited the analysis to participants' first visits. For a subset of the participants (50.3%), plasma‐based glial fibrillary acidic protein (GFAP), tau, and neurofilament light chain (NfL) measurements were available using the Quanterix assay. We investigated the association between depression, AD, and these three plasma biomarkers.

**Result:**

Among the 3,670 participants, the mean age was 70.6 years, with 61.7% being female and 36.0% identifying as Hispanic origin. Of the total sample, 38.5% were diagnosed with AD. Recent depression (defined as depression within two years prior to visit) was significantly associated with AD after adjusting for age, sex, self‐reported ethnicity and race (OR=2.50, *p* = 1.33x10^‐20^ ). The total score on the 30‐item GDS scale also showed a significant association with AD (OR=1.05, *p* = 1.25×10^−9^). Analysis of plasma biomarkers revealed significant associations between total GDS‐30 scores and three key markers: GFAP levels (β=0.0005, SD=0.0001, *p* = 1.16×10^−3^), total tau (β=0.003, SD=0.001, *p* = 1.25×10^−3^), and NfL levels (β=0.001, SD=0.0002, *p* = 2.10×10^−6^).

**Conclusion:**

Our findings demonstrate significant associations between depression, AD diagnosis, and key plasma biomarkers of neurodegeneration. The strong relationship between recent depression and AD diagnosis, along with the correlations between GDS‐30 scores and plasma biomarkers (GFAP, tau, and NfL), suggests potential shared neurobiological mechanisms between depression and AD pathology. To further validate these findings and explore potential demographic influences, we will present results from an additional independent cohort and conduct stratified analyses across sex and ethnic groups.

